# High-rate quantum key distribution with compact state preparation and detection

**DOI:** 10.1073/pnas.2521590123

**Published:** 2026-04-30

**Authors:** Guan-Jie Fan-Yuan, Wei-Xin Xie, De-Yong He, Xiao-Juan Huang, Shuang Wang, Zhen-Qiang Yin, Wei Chen, Qiong Li, Hao-Kun Mao, Guo-Wei Zhang, Yu-Long Wang, Guang-Can Guo, Zheng-Fu Han

**Affiliations:** ^a^Chinese Academy of Sciences Key Laboratory of Quantum Information, University of Science and Technology of China, Hefei 230026, China; ^b^Chinese Academy of Sciences Center for Excellence in Quantum Information and Quantum Physics, University of Science and Technology of China, Hefei 230026, China; ^c^Hefei National Laboratory, University of Science and Technology of China, Hefei 230088, China; ^d^School of Cyberspace Science, Faculty of Computing, Harbin Institute of Technology, Harbin 150000, China

**Keywords:** quantum key distribution, single-photon detector, integrated optics, quantum information, quantum optics

## Abstract

Quantum key distribution (QKD) is a promising technology for information-theoretic secure communications. The practicality of QKD systems is critical for large-scale network deployment, requiring both high key generation rate and miniaturized modules for quantum-state preparation and detection. Here, we report a deployable QKD system that achieves secure key rates of 60.33 ± 0.03 Mbps at 10 km and 3.08 ± 0.20 Mbps at 100 km. The system integrates a dual-parallel Mach–Zehnder encoder to achieve high-fidelity, unified preparation of polarization and decoy states via single-step modulation in a compact design. Meanwhile, we developed miniaturized single-photon avalanche detectors operating at 2.5 GHz, achieving high count rates while maintaining low noise. This high-performance and compact solution establishes a feasible roadmap for urban secure communication networks that require high capacity and bandwidth.

## Significance

Practical quantum communication demands high performance without reliance on bulky or cryogenic hardware. We propose a single-step polarization- and intensity-encoding scheme together with an efficient semiconductor-based single-photon detection for quantum information processing, and demonstrate quantum key distribution (QKD) over 100 km with a state-of-the-art secure key rate using a nonsuperconducting system. These results mark a key turning point, demonstrating that record-level performance can be achieved without expensive and sophisticated cryogenic infrastructure. Beyond QKD, the state preparation and detection mechanisms developed here are broadly applicable to other quantum technologies, including quantum computing and scalable quantum networks, thereby establishing a paradigm for the practicality of high-performance quantum information systems.

Quantum key distribution (QKD) (1, 2) is a technology for cryptographic information processing that exploits quantum resources, enabling the sharing of cryptographic keys with information-theoretic security. Combined with the one-time pad (OTP) (3), it enables secure communication whose security is independent of computational complexity. Key generation rate and system compactness determine QKD application boundaries. First, QKD distributes symmetric keys, the real-time key generation rate using OTP should not be lower than the classical communication bandwidth. Also, QKD needs to eliminate bulky quantum information processing equipments to enable practical deployment and enhance user accessibility.

The real-time performance of practical high-rate QKD systems is 10 Mbps at 10 km (4). To cover cities and metropolitan areas, the range should be extended to 50 to 100 km while maintaining a key rate of 3 Mbps for typical HD video conferencing (5). For ultra-high-definition video, Virtual Reality, and cloud gaming, key rates need to reach 25 to 50 Mbps (6).

Based on common decoy-state (7, 8) and finite-key method (9, 10), the secure key rate of a QKD system is primarily affected by factors such as repetition rate, error rates, and transmittance. Currently, the repetition rate of high-performance systems has reached the GHz level, with an error rate of only a few per cent or even lower (4, 11, 12). Over a 10 km fiber channel, the key rate has reached 110 Mbps using polarization encoding (13) and 60 Mbps with time-bin encoding (14).

Recent advancements in key rates primarily stem from the superior performance of superconducting detectors. Unfortunately, their requirements for ultra-low temperatures (∼K) and vacuum conditions imposes significant limitations on size and maintenance, hindering the achievement of large-scale general user accessibility for this technological approach (15). Similar challenges arise at the transmitter side. QKD systems not only prepare BB84 states across encoding dimensions (e.g., polarization, phase) but also require intensity modulation for decoy states. To address the requirements of intensity modulation, several MZ-based schemes have been proposed that exhibit good stability even in the presence of modulation-signal offsets (16–19). However, high-rate systems still employ a two-step separate modulation scheme for encoding and decoy states, increasing complexity and compromising compactness. On the other hand, on-chip integration can significantly reduce physical size (13, 20–24), it does not address scheme limitations, potentially impacting stability and yield.

In this paper, we present a practical high-rate QKD system featuring compact state preparation and detection. At the encoding side, we designed a dual-parallel Mach–Zehnder (MZ) structure-based scheme to prepare polarization and intensity simultaneously in a single step. It was also integrated to achieve compactness in both architecture and fabrication, demonstrating a misalignment error below 0.4%. On the detection side, we developed palm-sized avalanche photodiode single-photon detectors with advanced control strategies, achieving a 1.9% afterpulse probability at 40% detection efficiency and a saturation count rate of 200 MHz. Coupled with efficient error correction and postprocessing, this 2.5 GHz practical system achieves a real-time secure key rate of 60.33±0.03 Mbps at 10 km, extending the 10 Mbps key rate range beyond 50 km. Furthermore, at 100 km, it demonstrates the state-of-the-art key rate of 3.08±0.20 Mbps, which is sufficient to HD video communication.

Table 1 presents a comparison of this work with recent works in terms of encoder, detector, and key rate for 10 km and 100 km channels. The result significantly improves the key rate of practical QKD systems based on avalanche detectors. Meanwhile, the lightweight design supports a wider range of applications and lowers the barrier for users. It lays the foundation for establishing high-speed metropolitan networks with large user capacity.

**Table 1.** Comparison of high-rate QKD experiments

3-4 Reference	Detector	Key Rate (Mbps)	
		@10 km	@100 km
2018 (4)	InGaAs	13.72*	—
2023 (13)	SNSPD	115.8*	2.3*
2023 (14)	SNSPD	64	3.0
This work	InGaAs	60.33	3.08

These results were obtained using standard fiber with an attenuation of 0.2 dB/km.

## Experimental Setup

Our system is developed based on the standard decoy-state BB84 protocol and finite-key analysis (7–10). The state preparation and measurement of the BB84 protocol are simple, requiring only one sender (Alice) and one receiver (Bob). The decoy-state method further eliminates the need for single-photon sources, making it widely used in practical systems. Meanwhile, the decoy-state BB84 protocol has a well-established security proof considering finite-key effects, providing a solid foundation for achieving a high secure key rate. In this protocol, four BB84 states need to be prepared, which consist of two mutually unbiased basis eigenstates, $\sigma_{Z}$ and $\sigma_{X}$. The decoy-state method also requires intensity modulation to extract single-photon information using weak coherent states. For the measurement, both Z and X are required, where Z is used for key generation and X is used for error estimation. For the decoy-state protocols, we adopted the 1-decoy version (25). Generally, reducing the number of decoy states results in better performance in finite-key scenarios, but it worsens the estimation of error rate. Under the system parameters of low misalignment error and large finite-key, the 1-decoy scheme achieves a higher simulated key rate. The detailed analysis is provided in *SI Appendix*, *Choice of Decoy-State Scheme*.

**Fig. 1.**
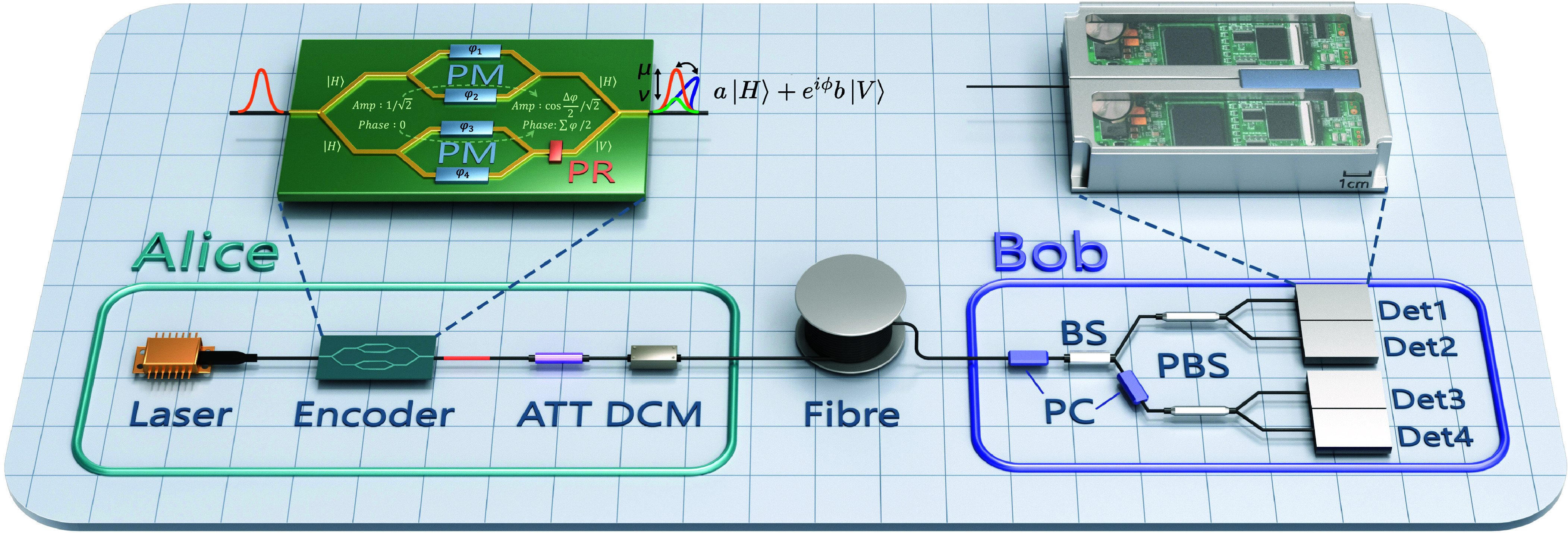
Schematic diagram of the experimental setup. The detailed enlarged diagram of the encoder and detector is provided. The encoder employs parallel Mach–Zehnder (MZ) structures, in which the amplitude and phase of the optical pulses are independently modulated after passing through each MZI, with the amplitude determined by the phase sum and the phase determined by the phase difference. The upper and lower paths correspond to horizontal and vertical polarizations, respectively, enabling the preparation of arbitrary quantum states. PM, phase modulator; PR, polarization rotator; ATT, attenuator; DCM, dispersion compensation module; PC, polarization controller; BS, beam splitter; PBS, polarization beam splitter; Det1-4, four single-photon detectors, with two fabricated together to form one device.

To meet the requirements of the protocol while balancing high speed and compactness, we designed the experimental system shown in Fig. 1. The Alice side consists of a gain-switched laser and an encoder. The laser emits 1,550 nm pulses with a full width at half maximum of 30 ps at a repetition rate of 2.5 GHz. The pulses are modulated by the encoder into different polarization states and decoy states. An attenuator (ATT) reduces the pulse power to the single-photon level. A dispersion compensation module (DCM) is utilized to mitigate pulse broadening caused by channel dispersion. These quantum states travel through ultra-low-loss fiber (0.158 dB/km) to reach Bob’s side.

Bob uses a passive basis selection, where a custom unbalanced beam splitter (BS) guides the incoming pulses to the decoding devices. Each decoding part consists of a polarization controller (PC), a polarization beam splitter (PBS), and two gated detectors (DET). The PC is responsible for aligning quantum states to the Z basis or X basis, while the PBS separates the two polarization states of the basis. By selecting a fixed ratio for the unbalanced BS and using fully fused connections, the insertion loss at the receiver side is approximately 0.3 dB. The detectors perform single-photon detection at a gating rate of 2.5 GHz with a gate width of 100 ps (full width at half maximum).

The encoder is a core component because its polarization and intensity modulation capabilities establish the feasibility of implementing the protocol. Here we utilize a dual-parallel MZ structure based on LiNbO_3_ to modulate quantum states, as shown in Fig. 1. The incoming pulses are first split into two equal parts and then enter the upper and lower MZ structures, respectively. Each arm of the Mach–Zehnder interferometer (MZI) has a phase modulator. After passing through the interferometer, the intensity of the pulses depends on the difference of modulating phases, while their phase depends on the sum of the modulating phases. Then the output pulses of one MZI are rotated by 90 degrees in polarization and output together with the pulses of the other MZI.

In this design, the two MZIs are essentially responsible for preparing |H〉 and |V〉, respectively. Since each MZI can modulate intensity and phase, it is allowed to prepare a state of $a|H\rangle +e^{i\phi }b|V\rangle$, where a and b (a,b≥0) represent the intensities of the orthogonal components, and ϕ represents the relative phase. Due to the path difference between the two MZIs and the dispersion of the polarization-maintaining (PM) pigtails, a delay of 3.9 ps is introduced between the fast-axis pulse and the slow-axis pulse. A 3-m-long PM fiber, the orange segment shown in the figure, is connected to the pigtail via a 90-degree flange to compensate for the delay. The test results indicate that the isolation of the MZIs can reach 27 and 23 dB in |H〉 and |V〉, corresponding to the misalignment errors of 0.19% and 0.49%. More detailed information is provided in *Materials and Methods* and *SI Appendix*, *Encoding Principle*.

Single-photon detectors are equally critical for high-performance QKD systems. Based on our sinusoidal-gating technology (26) and two stages band-stop filtering, the discrimination sensitivity of avalanche signals is significantly enhanced, enabling higher detection efficiency and lower noise. Using this approach, four 2.5-GHz InGaAs single-photon detectors were fabricated, achieving a detection efficiency of 40% and a saturation count rate of 200 MHz, while maintaining an afterpulsing probability below 2% and a dark count rate below $1.25\times 10^{-5}$. The detection efficiency and noise level both impact the key rate. Under the 10 km benchmark, detection efficiency plays a dominant role, whereas, for longer distances, the key rate becomes more sensitive to noise (11, 27). Here, we adopted the optimization strategy from ref. 28, increasing the amplitude of the gating signal to 15 Vpp and raising the temperature of the avalanche diode to 0 ^°^C, in exchange for an improvement in detection efficiency. At the same time, the temperature increase helps reduce afterpulsing. Although the elevated temperature also increases the dark-count rate, its impact on the key rate under the short-distance benchmark is minimal. On the other hand, there is a trade-off between dead time and afterpulsing. A longer dead time can reduce afterpulsing but can impact the counting rate. We also optimized the dead time to 5 ns to obtain a higher key rate. The method for achieving temperature independence is described in *Materials and Methods*. *SI Appendix*, *Detector Performance* provides more detailed information on the test results of the detectors.

For data acquisition, we utilize the SIMINICS MT16 time-to-digital converter (TDC), which features a time resolution of 1 ps, a dead time of 2 ns, and a saturation count rate of 500 Mcps. To ensure real-time processing, we employ a fiber Ethernet interface to download data at a speed of 280 Mcps. The raw keys are processed for basis sifting, error correction, and privacy amplification on an Intel Core i9-14900K platform. Error correction is performed using a multithreaded and block-optimized Cascade protocol (29), achieving a throughput of 248 Mbps per instance with an efficiency of 1.03. Privacy amplification is implemented using modular arithmetic hash accelerated by the GNU Multiple Precision Arithmetic Library (30), achieving a throughput of 174 Mbps per instance with a block length of 2,147,352,576 (≈2$\times \;10^{9}$) bits. The postprocessing section adopts a multithreaded pipeline design, with multiple instances of each module running concurrently.

## Implementation

Based on the aforementioned system, we conducted QKD experiments over channel lengths ranging from 10 to 50 km, with intervals of 10 km. Notably, the actual optical fiber channel length is fixed at 60 km to match the dispersion compensation. We achieve an equivalent shorter channel length by reducing the attenuation of ATT, resulting in harsher experimental conditions in terms of channel disturbances for shorter distances. With the security parameters of $\epsilon_{\sec}=10^{-10}$ and $\epsilon_{\mathrm{corr}}=10^{-15}$, we optimize the intensity and probability of signal and decoy states, $\mu ,\nu ,P_{\mu },P_{\nu }$, as well as the basis selection probability, $P_{Z}$, for each distance. A comprehensive detector model that takes into account dead time and afterpulsing is utilized for accuracy. The optimized parameters are then used to generate random sequences to drive the encoder.

To further reduce the error rate, we optimize both timing control and data processing. Conventionally, optical pulses are aligned with the temporal position of maximum detection probability within the detector gate. Here, we intentionally introduce a slight delay in the arrival time of the optical pulses relative to this position. This timing shift shortens the effective avalanche duration, as the pulse arrival becomes closer to the gate-closing time, thereby reducing the probability of afterpulsing. This strategy comes at the cost of a moderate reduction in detection efficiency, reflecting an inherent trade-off between afterpulsing suppression and detection efficiency.

In addition, although the optical pulses are centered within the 400-ps gating period, the time-to-digital converter records detector click events with a temporal distribution spanning the entire period. Events occurring near the edges of the gating window predominantly originate from detector noise or residual signals from the preceding gate, and therefore contribute to the quantum bit error rate. By applying temporal windowing to the event timestamps and discarding events near the gate edges, we effectively suppress error contributions from these noise-dominated events.

After optimization, we select a 10 ps delay for the optical pulse and define a time window from 48 ps to 368 ps. As a result, the quantum bit error rate (QBER) in Z basis can decrease by 16.7%, accompanied by a 5.6% decrease in detection efficiency. Detailed results can be found in *SI Appendix*, *Detector Performance*.

**Fig. 2.**
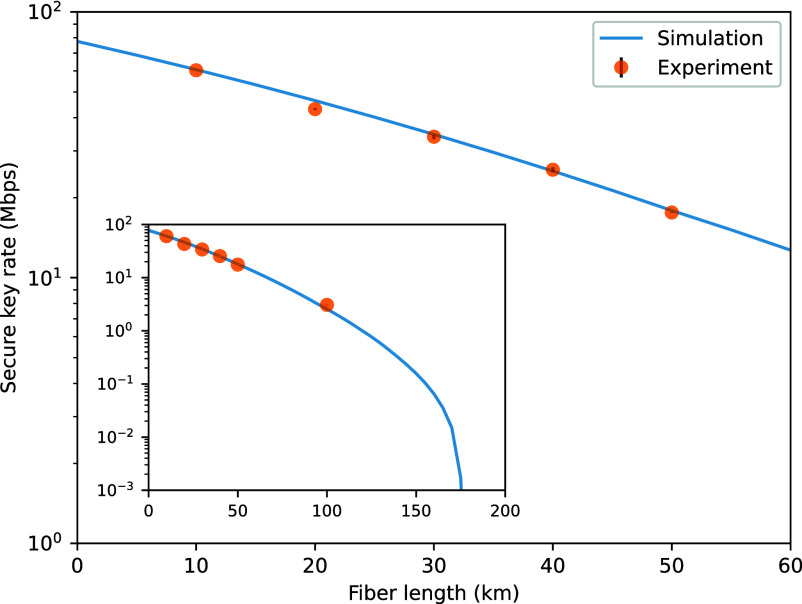
Experimental and simulated secure key rates.

At a distance of 10 km, we achieved a secure key rate of 60.33±0.03 Mbps. Correspondingly, the average bit error rate is 1.59% and the average error correction efficiency is 1.03. For distances of 20, 30, 40, and 50 km, the corresponding secure key rates are 43.08±0.36, 33.91±0.72, 25.48±0.58, and 17.59±0.07 Mbps. At 100 km, the key rate becomes more sensitive to errors. Detector parameters are adjusted to reduce dark count rate and afterpulse probability at the cost of detection efficiency, achieving a 3.08±0.20 Mbps key rate. Fig. 2 presents experimental secure key rates at various distances, compared with the simulation curve. Each experimental point is marked with a circle, and SE bars obtained from multiple measurements are also included. For more detailed data, see *SI Appendix*, *Experimental Data*.

**Fig. 3.**
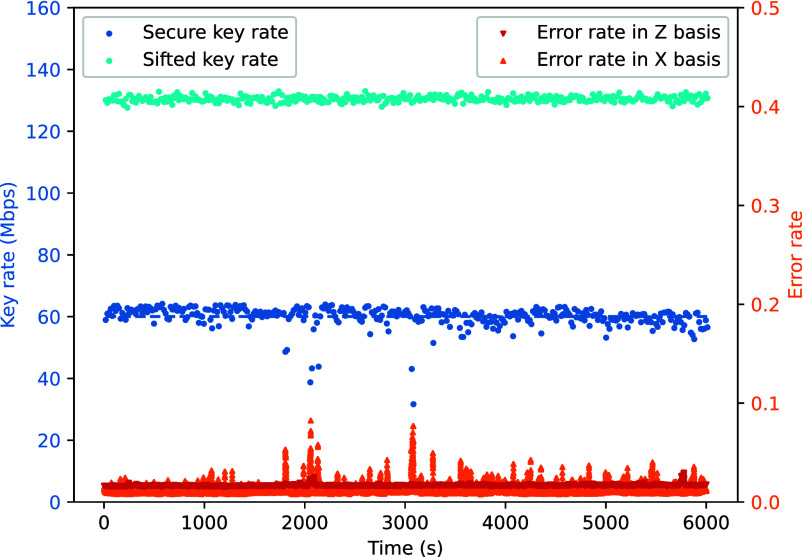
Key generation in continuous operation. The cool-colored circles represent key rate, corresponding to the left Y-axis, and the warm-colored triangles represent error rate, corresponding to the right Y-axis.

Fig. 3 shows the key rates and error rates during continuous operation over 10 km of fiber, corresponding to the condition where the data-flow load on the system is the highest. The left vertical axis represents the key rate, while the right vertical axis represents the error rate. The data record 363 rounds of key generation, corresponding to a continuous running over 6,000 s. The key rates are recorded every round, while the error rates (also known as QBER) are updated every 0.3 s to capture more details during the operation, resulting in a denser set of data points.

The key rate generally remains stable. The sifted key rate is related to the counting rates, reflecting the stability of the transmitter’s intensity modulation and the receiver’s detector performance. The secure key rate is also affected by error rates, showing larger fluctuations. When estimated using a normal distribution, the mean is 60.03 Mbps with a SD of 3.41 Mbps. The fluctuations mainly arise from channel disturbances and the imperfect compensation of these disturbances. The experimental system operated in an open laboratory environment with no control of temperature, humidity, vibration, or cleanliness. As the transmission channel, the fiber spools were susceptible to vibration and temperature variations, which induced perturbations in both delay and polarization and ultimately manifested as data fluctuations. The error rate in the X basis fluctuates more than that in the Z basis, primarily because the amount of X-basis data is much smaller than that of the Z basis, introducing additional statistical fluctuations and further influencing the effectiveness of polarization compensation. Moreover, the X basis is affected by both intensity and relative-phase fluctuations, whereas the Z basis is only influenced by intensity fluctuations. Overall, although the open experimental environment introduced nonnegligible disturbances, the system was able to autonomously recover from these perturbations and maintain continuous operation, demonstrating strong robustness.

## Discussion

In conclusion, we realize a high-speed QKD system with a secure key rate exceeding 60 Mbps at 10 km and 3 Mbps at 100 km. The system’s integrated encoder and high-performance InGaAs single-photon detectors contribute to its compactness and ease of maintenance, enhancing its practicality. The adoption of polarization encoding demonstrates the advantages of our modulation design and allows for a low-loss receiver, offering a complementary alternative to phase-based systems. On this foundation, we optimize detector parameters using an afterpulse-dead-time model and APD regulation strategies. Additionally, we reduce error rates through temporal filtering and shifting and realize a high-throughput and low-key-loss postprocessing module, further improving the key rate.

Although the key generation at short distances is less sensitive to errors compared to long distances, the bottleneck for the system’s key rate remains the error rate, as the detector count rate and data processing speed are already sufficient. The primary source of errors is the detector’s afterpulsing, which is also a major challenge for high-performance practical QKD systems. This is because an increase in the count rate necessitates a reduction in dead time, which exacerbates the afterpulsing effect. Therefore, achieving lower noise detection is key to improving the key rate in the future. For key generation at longer distances, dark counts become the dominant limiting factor. This is because afterpulsing, as a correlated response, decreases proportionally with the photon count rate, whereas dark counts constitute a constant noise floor whose relative impact grows as the channel loss increases. In networks with varying channel conditions, the detector-parameter adjustment strategy adopted in this work allows the detector to operate in different performance modes, providing adaptability to diverse channel environments. Nevertheless, such adjustments offer only limited optimization space. To pursue significantly higher performance, further improvements in the fundamental detector characteristics, particularly suppressing dark counts while maintaining high detection efficiency, will be necessary. Such advancements would further amplify the advantages of semiconductor detectors in practical deployment scenarios and allow them to complement superconducting detector systems, which have inherent strengths at extreme distances.

Our result is an advancement for the field of practical applications. In short-distance scenarios, the key rate reaches a level suitable for modern internet applications. More importantly, the practical characteristics of our system make it more aligned with the needs of large-scale deployment, particularly in metropolitan area networks, which are densely populated with users. At the same time, the results for long distances also suggest that to meet the requirements of more wide-area scenarios, dynamic optimization of the detectors is necessary as the key rate becomes more sensitive to noise. For field deployment, the open experimental environment captures similar types of disturbances, and practical loss and coexistence effects can be reliably estimated, indicating that the proposed improvements hold strong promise in real-world use. Therefore, this result will effectively accelerate the practical implementation of high-performance QKD, thereby facilitating the construction of information-theoretic secure communication networks and providing a development paradigm for the practicality of high-performance quantum information systems.

## Materials and Methods

### State Preparation.

Intensity and polarization modulation are implemented using the four phase modulators on the two MZIs. For each MZI, the output intensity depends on the phase difference, while the output phase is determined by the phase sum. By tuning the phase difference we control the intensity, and by tuning the phase sum we control the relative phase between the two MZIs, enabling polarization-state preparation. To determine the electrical levels required for generating different quantum states, we first align the signal delays of the four phase modulators and drive each MZI to its off state to define the intensity reference. We then calibrate the mapping between electrical levels and intensity by adjusting one of the two electrical levels applied to the MZI, and determine the mapping for phase by equalizing the two MZI intensities and synchronously varying the electrical levels applied to the two arms of one MZI while keeping its intensity constant. A more detailed discussion is provided in *SI Appendix*, *Encoding Principle*.

### Detector Temperature Independence.

The detector performance is designed to remain stable under environmental temperature variations. Temperature variations mainly influence the breakdown voltage of the single-photon avalanche diode and the gain of the peripheral amplifiers. The single-photon avalanche diode is actively stabilized by a thermo-electric cooler with a temperature precision better than 0.01 ^°^C, and within the typical laboratory range (approximately ±5 ^°^C), the gain variations of the amplifiers are limited, leading to only a small influence on the overall detector performance. For wider temperature variations, compensation was applied to the amplifier gains, which can keep the relative fluctuations in detection efficiency, dark count rate, and afterpulse probability below 1%.

## Supplementary Material

Appendix 01 (PDF)

## Data Availability

Data to support all figures are available under the following FigShare Repository (https://doi.org/10.6084/m9.figshare.29652746) (31). All other data are included in the manuscript and/or supporting information.
