# Supplementary material for: High-rate quantum key distribution with compact state preparation and detection
Source: Proc Natl Acad Sci U S A. 2026 Apr 30;123(18):e2521590123. doi: 10.1073/pnas.2521590123 (PMC13143002; doi:10.1073/pnas.2521590123)
Supplement: Supplementary file 1 — Appendix 01 (PDF) [file pnas.2521590123.sapp.pdf]

Supporting Information for

High-rate quantum key distribution with compact state preparation and detection

Guan-Jie Fan-Yuan, Wei-Xin Xie, De-Yong He, Xiao-Juan Huang, Shuang Wang, Zhen-Qiang Yin, Wei Chen, Qiong Li, Hao-Kun Mao, Guo-Wei Zhang, Yu-Long Wang, Guang-Can Guo and Zheng-Fu Han

Shuang Wang
E-mail: wshuang@ustc.edu.cn

This PDF file includes:

Supporting text
Figs. S1 to S4
Tables S1 to S3
SI References

Supporting Information Text

Choice of Decoy-State Scheme

To evaluate the performance of decoy-state methods, we first establish a system model, then simulate and optimise the key rate using different decoy-state protocols, and finally select the optimal parameters as the experimental settings.

There are four states prepared by Alice, which are combinations of two bases ($B = \{Z, X\}$) and two keys ($K = \{0, 1\}$). Bob also has four detectors, each dedicated to measuring one of these four states. Taking the Z_0 detector as an example, we present the classical response model when transmitting the four different states(1).

$$\begin{aligned} Q^{Z_0}_{\alpha,Z_0} &= 1-(1-Y_0)e^{-P_Z\alpha\eta(1-e_d)}, \\ Q^{Z_0}_{\alpha,Z_1} &= 1-(1-Y_0)e^{-P_Z\alpha\eta e_d}, \\ Q^{Z_0}_{\alpha,X_0} &= 1-(1-Y_0)e^{-P_Z\alpha\eta/2}, \\ Q^{Z_0}_{\alpha,X_1} &= 1-(1-Y_0)e^{-P_Z\alpha\eta/2}, \end{aligned} \quad [1]$$

where the superscript of Q represents the Z_0 detector, and the subscript represents the transmitted state. $\alpha \in \{\mu, \nu, \omega\}$ is the mean photon number of the coherent state, Y_0 is the dark-count rate, P_Z is the probability of Bob selecting Z basis, η is the total transmittance including channel loss, insertion loss, and detection efficiency.

The first two and the last two formulas represent the cases where Alice and Bob select the same or different bases, respectively. The first and third, and the second and fourth formulas represent the cases where their keys are the same or different, respectively. The response formulas for the other three detectors are similar. Below, we continue using the Z_0 detector as an example for afterpulsing and dead-time correction.

According to Ref.(2), when considering afterpulsing, the response rate model becomes

$$\hat{Q}^{Z_0}_{\alpha,\beta} = 1-(1-Q^{Z_0}_{\alpha,\beta})(1-P_{ap}), \quad [2]$$

where $\beta = \{Z_0, Z_1, X_0, X_1\}$ represents the transmitted state, and P_{ap} is the overall afterpulse probability. Further, the correction model for dead-time is given by Ref. (3)

$$\tilde{Q}^{Z_0}_{\alpha,\beta} = \frac{\hat{Q}^{Z_0}_{\alpha,\beta}}{1+\tau\sum_{\alpha,\beta} P_\alpha P_\beta \hat{Q}^{Z_0}_{\alpha,\beta}}, \quad [3]$$

where τ represents the number of invalid gating caused by the dead time. For a dead time of 5 ns and a gate frequency of 2.5 GHz, this corresponds to $\tau = 12$.

At this point, we can present the gain and error rate under Z basis as

$$\begin{aligned} Q^Z_\alpha &= \tilde{Q}^{Z_0}_{\alpha,Z_0} + \tilde{Q}^{Z_0}_{\alpha,Z_1}, \\ EQ^Z_\alpha &= (1-\tilde{Q}^{Z_0}_{\alpha,Z_0})\tilde{Q}^{Z_0}_{\alpha,Z_1} + \frac{1}{2}\tilde{Q}^{Z_0}_{\alpha,Z_0}\tilde{Q}^{Z_0}_{\alpha,Z_1}. \end{aligned} \quad [4]$$

Based on these models and the existing decoy-state methods(4, 5), we can obtain the key rate. For example, the key rate using 1-decoy method is given by

$$\begin{aligned} l \leq & s^l_{Z,0} + s^l_{Z,1}(1-h(\phi^u_Z)) - f n_Z h(E_Z) \\ & - 6\log\left(\frac{19}{\epsilon_{sec}}\right) - \log\left(\frac{2}{\epsilon_{corr}}\right), \end{aligned} \quad [5]$$

where $h(x)$ is the binary entropy function, f is the error correction efficiency, ϕ^u_Z is the upper bound of the phase error rate in Z basis, E_Z is the quantum bit error rate in Z basis, $s^l_{Z,0}$ and $s^l_{Z,1}$ represent the lower bounds for the vacuum state and single-photon counting, respectively. The upper bounds and lower bounds can be obtained by experimental observable quantities Q and E using the decoy-state method in (4, 5).

Figs.S1 demonstrates the impact of channel length on the secure key rate. As the number of decoy states increases, the parameter estimation becomes more precise, but it is opposite under the finite-key effect. At shorter distances, the count rate is higher and the error rate is lower. Therefore, the disadvantage of the 1-decoy-state method is not as apparent, and its advantages are emphasized, resulting in a higher key rate.

Encoding Principle

The encoder is based on a dual parallel Mach-Zehnder interferometer. Fig.S2 illustrates the principle diagram, serving as an equivalent optical path for analysis.

The principle of this structure is to independently modulate the intensity and phase of the horizontal and vertical polarization states and then combine them using a PBS, thereby preparing arbitrary polarization states. The incident optical pulse is first split into two parts by BS1, corresponding to the horizontal and vertical polarization states. Subsequently, the intensity and phase of each polarization state are modulated by an MZ structure. Finally, the pulses are recombined and output.

We implemented the above encoding scheme using mature titanium-diffused lithium-niobate waveguide integration technology. A symmetric Y-branch provides stable power splitting, a micro-assembled crystalline rotator performs precise 90° polarization rotation, and a film-type PBS enables low-loss polarization combining. After optoelectronic packaging, the overall module size is approximately $1 \times 8\,\mathrm{cm}^2$.

Fig.S2 also labels the expressions of the quantum states at different stages. The intensity coefficient of the horizontal polarization is $\left|\frac{1}{\sqrt{2}}\cos\left(\frac{\phi_1-\phi_2}{2}\right)\right|^2$ and phase is $\frac{\phi_1+\phi_2}{2}$. The intensity coefficient of the vertical polarization is $\left|\frac{1}{\sqrt{2}}\cos\left(\frac{\phi_3-\phi_4}{2}\right)\right|^2$ and phase is $\frac{\phi_3+\phi_4}{2}$. Therefore, by turning the MZI on and off, the preparation of horizontal and vertical ($|H\rangle$ and $|V\rangle$) polarization states can be achieved. By ensuring the intensity coefficients of the two MZIs are the same and adjusting the phases, the preparation of $\pm 45°$ ($|D\rangle$ and $|A\rangle$) and left-hand/right-hand circular ($|L\rangle$ and $|R\rangle$) polarization states can be realized. Let the incident light intensity be α, the output light intensity be $\mu = a\alpha$, where a is the intensity coefficient. According to the phase modulation in Tab.S1, the six states with a given intensity can be prepared.

In the experiment, we use $|H\rangle$ and $|V\rangle$ to form the Z basis, and $|D\rangle$ and $|A\rangle$ to form the X basis. The specific polarization states and intensities are prepared according to the quantum-random-number sequence, which is processed using the optimized parameters and translated into the corresponding modulation settings of our encoding scheme. The system operates in a multi-producer–multi-consumer architecture, where a ring-buffer mechanism is used to bridge the data-rate differences between upstream and downstream processes, enabling efficient and continuous system operation. The misalignment error rate, e_d, depends on the extinction ratio of the MZ. The measured extinction ratios are 27.3 dB (0.19% error) and 23.1 dB (0.49% error), corresponding to a total misalignment error rate below 0.4%.

Detector Performance

Unlike superconducting detectors, there are more factors influencing the performance of avalanche photodetectors. The basic parameters of APDs, detection efficiency (η_d), afterpulsing probability (P_{ap}), and dark-count rate (Y_0), are all affected by factors like discriminator level, bias voltage, temperature, and dead time, creating interdependencies. This complex relationship makes it challenging to optimize the detectors. To address this, we anchor the detection efficiency at 40% and adjust other parameters to minimize the afterpulsing probability. Since dark counts have a relatively small impact on the secure key rate, they are not considered in this optimization.

The testing of the detector followed standard procedure(6, 7). We use 20 MHz coherent state pulses with an intensity of 0.05 to calibrate the detection efficiency and afterpulse probability. The dark counts are collected in the absence of optical pulses. Tab.S2 provides the basic parameters of the detector.

However, during actual operation, the afterpulsing probability tends to increase, which is related to the principles of dead time. There are two types of dead time. One is the true dead time, during which the detector cannot ignite an avalanche. The other is the false dead time, during which the detector can avalanche, but the avalanche signal is filtered out by the electronics. To ensure the quality of the gating signal, high-speed detectors cannot complete shutdown, therefore, the dead time scheme adopts the latter (false dead time). This means that avalanche signals occurring during the dead time contribute to afterpulsing, causing the afterpulsing probability to increase with the incident light intensity. Therefore, afterpulsing increases by approximately $Q \times \tau$, where Q is the count rate without dead time and τ is the number of gating filtered out by the dead time. For the 10 km parameters, with $Q_z = 0.044$ and $\tau = 12$, the afterpulsing increases by approximately 52.8%, which is consistent with our measured value of $P_{ap,10km} = 3.04\%$ at the intensity and probability parameters of 10km.

The additional afterpulsing caused by high intensity (or high counting rate) significantly increases the error rate. To reduce it, we adopt the delay deviation and time-window filter method. The former delays the arrival of the optical pulse at the detector, reducing the avalanche time and thus lowering afterpulsing. The latter filters out noise signals at the edges of the detection window. However, such operations also reduce the counting rate, which is equivalent to a decrease in detection efficiency.

We measured the afterpulse probability and detection efficiency of detectors for 11 different delays and 19 different time windows, resulting in 209 combinations, as shown in Fig.S3. Further simulations are performed to determine the optimal secure key rate, as depicted in Fig.S4. A time-window L+R means a window of $L \times 16$ ps $\sim R \times 16$ ps. The optimal point is marked by a red pentagram.

The combination yielding the highest key rate is selected as the experimental parameter. The delay is set to 10 ps, with the time window being 3+2, meaning that the signals in the first 48 ps and the last 32 ps of the cycle are filtered out. This configuration results in an afterpulsing probability of 2.22%, a detection efficiency of 37.75%, and a simulated secure key rate of 60.65 Mbps.

Experimental Data

This section provides the experimental parameters and data for five distances. At each distance, we optimized intensities and probabilities, and performed seven measurements for error estimation, corresponding to the data points shown in Fig. 2 of the main text. The parameters and results of one sample are presented in Tab.S3.

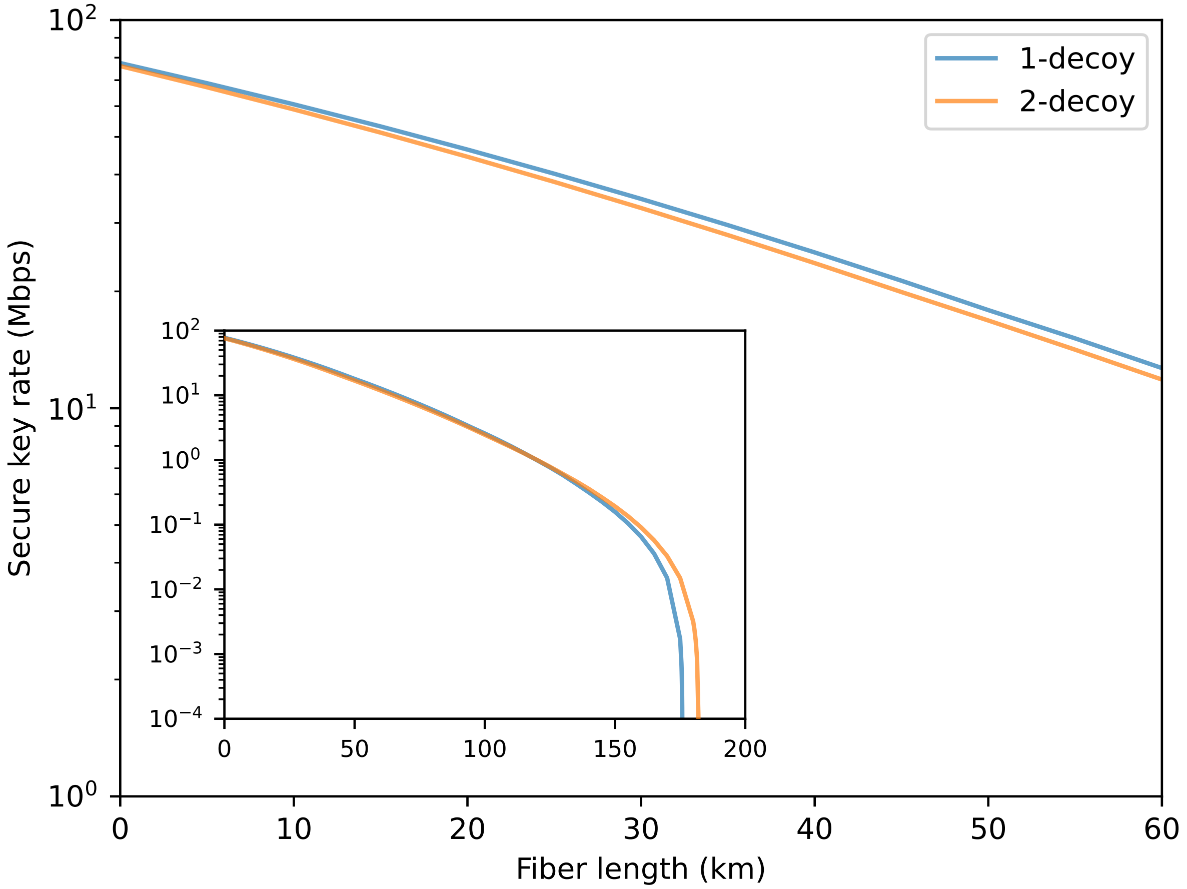

Fig. S1. Secure key rates versus fiber length using 1-decoy-state and 2-decoy-state methods.

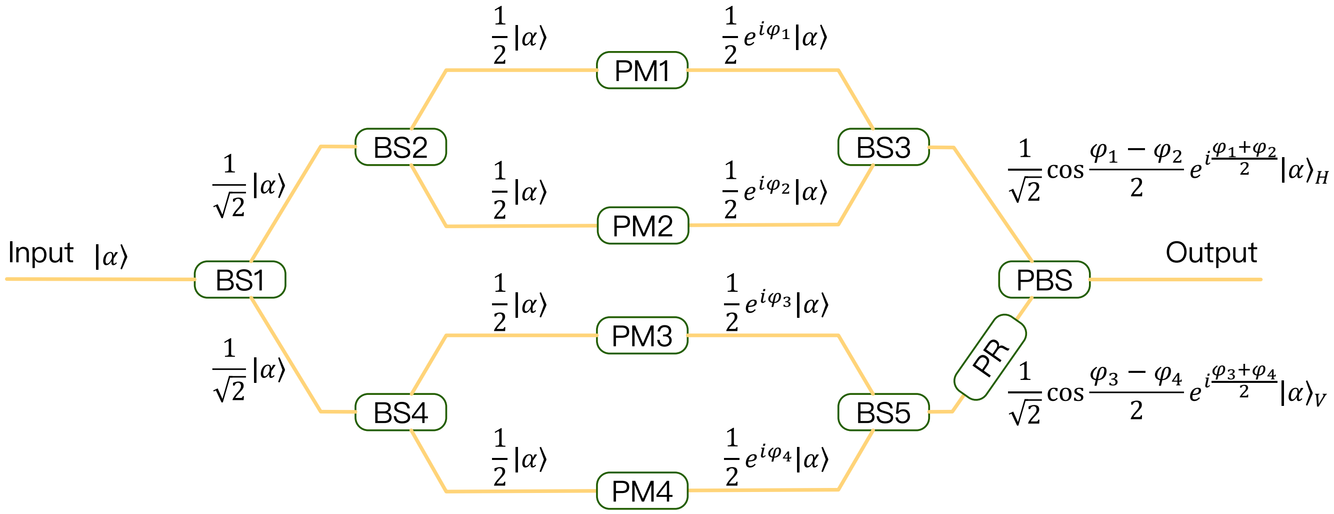

Fig. S2. Principle diagram of the encoder. BS, 50:50 beam splitter; PM, phase modulator; PBS, polarization beam splitter. The phases modulated by PM1 to PM4 are represented by ϕ_1 to ϕ_4, respectively.

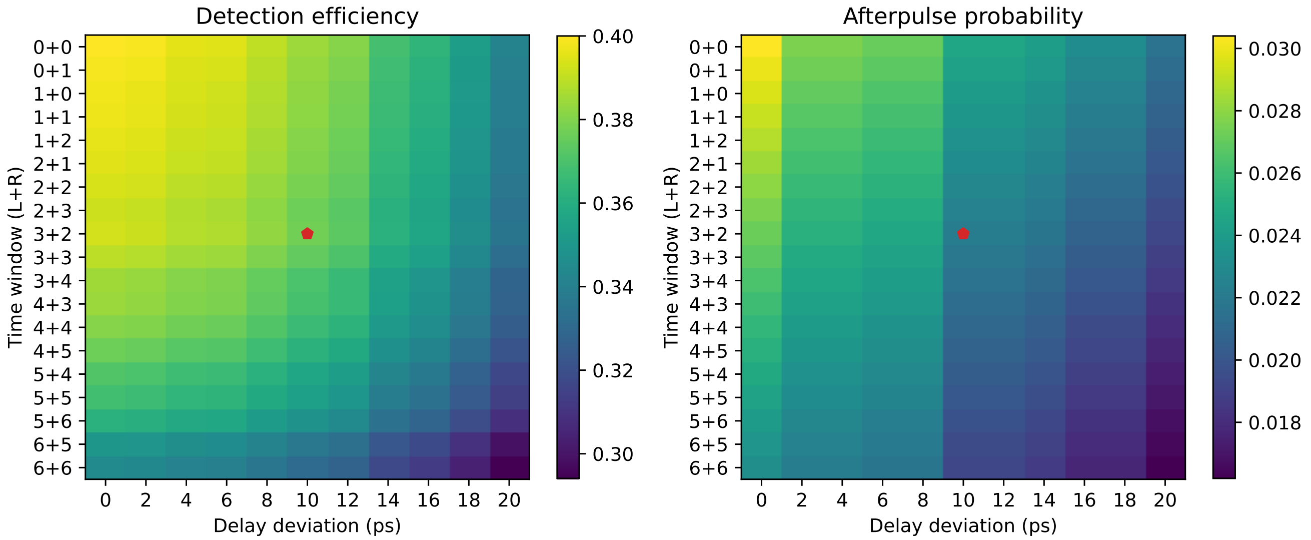

Fig. S3. Detection efficiency and afterpulse probability in different delay deviations and time-windows.

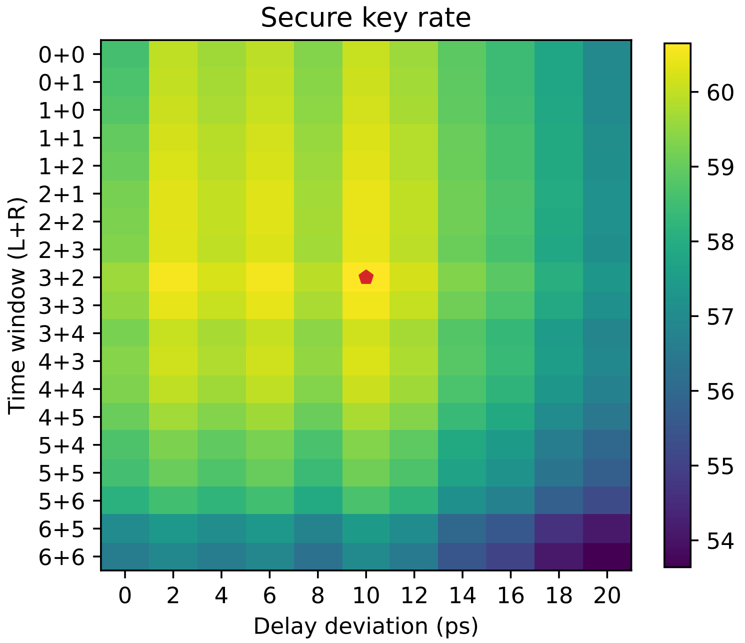

Fig. S4. Secure key rate in different delay deviations and time-windows.

Table S1. Encoding table

	ϕ_1	ϕ_2	ϕ_3	ϕ_4
$\lvert H\rangle_\mu$	$\mathrm{acos}\left(\sqrt{2a}\right)$	$-\mathrm{acos}\left(\sqrt{2a}\right)$	$\pi/2$	$-\pi/2$
$\lvert V\rangle_\mu$	$\pi/2$	$-\pi/2$	$\mathrm{acos}\left(\sqrt{2a}\right)$	$-\mathrm{acos}\left(\sqrt{2a}\right)$
$\lvert D\rangle_\mu$	$\mathrm{acos}\left(\sqrt{a}\right)$	$-\mathrm{acos}\left(\sqrt{a}\right)$	$\mathrm{acos}\left(\sqrt{a}\right)$	$-\mathrm{acos}\left(\sqrt{a}\right)$
$\lvert A\rangle_\mu$	$\mathrm{acos}\left(\sqrt{a}\right)+\pi$	$\pi-\mathrm{acos}\left(\sqrt{a}\right)$	$\mathrm{acos}\left(\sqrt{a}\right)-\pi$	$-\pi-\mathrm{acos}\left(\sqrt{a}\right)$
$\lvert L\rangle_\mu$	$\mathrm{acos}\left(\sqrt{a}\right)+\pi/2$	$\pi/2-\mathrm{acos}\left(\sqrt{a}\right)$	$\mathrm{acos}\left(\sqrt{a}\right)-\pi/2$	$-\pi/2-\mathrm{acos}\left(\sqrt{a}\right)$
$\lvert R\rangle_\mu$	$\mathrm{acos}\left(\sqrt{a}\right)+3\pi/2$	$3\pi/2-\mathrm{acos}\left(\sqrt{a}\right)$	$\mathrm{acos}\left(\sqrt{a}\right)-3\pi/2$	$-3\pi/2-\mathrm{acos}\left(\sqrt{a}\right)$

Table S2. Detector performance

Detector	η_d	P_{ap}	Y_0
Z_0	40%	1.9%	0.80×10^{-5}
Z_1	40%	2.0%	0.84×10^{-5}
X_0	40%	1.8%	1.28×10^{-5}
X_1	40%	1.8%	1.00×10^{-5}

Table S3. Experimental parameters and results

	10 km	20 km	30 km	40 km	50 km	100 km
kiloframe	33372	40619	52299	63222	83590	415871
time (s)	16.69	20.31	26.15	31.61	41.78	207.94
μ	0.436	0.455	0.475	0.491	0.500	0.582
ν	0.078	0.079	0.078	0.082	0.084	0.078
P_μ	0.88	0.88	0.88	0.88	0.88	0.92
P_Z	0.96	0.96	0.96	0.96	0.96	0.92
n_μ^Z	2138233098	2123008335	2142185023	2105103894	2125647145	2139788225
m_μ^Z	32482350	31588898	31120029	33341238	37898479	25908189
n_ν^Z	61089526	57374385	57465281	55618026	55259863	30567487
m_ν^Z	3798428	3540773	3108401	3125513	2975940	1574862
n_μ^X	5954285	5433547	5003562	4240567	5104093	17334066
m_μ^X	64428	68221	73155	69101	83206	536316
n_ν^X	149386	137076	132959	111122	111554	242953
m_ν^X	6372	6302	7261	7167	9076	38641
f_{EC}	1.03	1.03	1.03	1.03	1.03	1.03
l_s	2158573368	2174008548	2173393998	2160565230	2161780540	2157152886
l_f	1008643328	908746624	958231744	856831744	748087552	683339200
R (Mbps)	60.45	44.74	36.64	27.11	17.90	3.29

References

1. X Ma, B Qi, Y Zhao, HK Lo, Practical decoy state for quantum key distribution. *Phys. Rev. A* **72**, 012326 (2005).
2. GJ Fan-Yuan, et al., Afterpulse analysis for quantum key distribution. *Phys. Rev. Appl.* **10**, 064032 (2018).
3. XJ Huang, et al., Dependency model for high-performance quantum-key-distribution systems. *Phys. Rev. A* **106**, 062607 (2022).
4. CCW Lim, M Curty, N Walenta, F Xu, H Zbinden, Concise security bounds for practical decoy-state quantum key distribution. *Phys. Rev. A* **89**, 022307 (2014).
5. D Rusca, A Boaron, F Grünenfelder, A Martin, H Zbinden, Finite-key analysis for the 1-decoy state qkd protocol. *Appl. Phys. Lett.* **112**, 171104 (2018).
6. DY He, et al., 2.5 ghz gated ingaas/inp single-photon avalanche diode with 44 ps time jitter. *Adv. Devices & Instrumentation* **4**, 0020 (2023).
7. GJ Fan-Yuan, et al., Optimizing single-photon avalanche photodiodes for dynamic quantum key distribution networks. *Phys. Rev. Appl.* **13**, 054027 (2020).
